# Damage analysis and mechanism study of sol–gel coating over KDP crystal under multi-pulse of laser irradiation at low flux

**DOI:** 10.1038/s41598-022-25168-4

**Published:** 2023-03-01

**Authors:** Teng-Hui You, Wei Yang, Hao-Hao Hui, Xiang-Yang Lei, Tian-Yu Wang, Qing-Hua Zhang, Xin Ju, Xue-Ran Deng

**Affiliations:** 1 Department of Physics, University of Science and Technology Beijing, Beijing, 100083 People’s Republic of China; 2 Research Center of Laser Fusion, China Academy of Engineering Physics, Mianyang, 621900 People’s Republic of China

**Keywords:** Materials for optics, Nanoscale materials

## Abstract

The purpose of this study is to analyze the damage of antireflective (AR) coating over potassium dihydrogen phosphate (KDP) crystal subjected to multi-pulse laser irradiation at low flux under vacuum. Fresh silica AR was characterized as a reference; Atomic Force Microscope (AFM), Scanning Electron Microscopy (SEM), profilometer, and Scanning Near-Field Optical Microscope Photo-induced Force Microscope (SNOM-PiFM) were employed to analyze the characteristics of coatings. The experimental results indicated that the damage of AR coating over the KDP crystal was mainly caused by partial exfoliation, which exposed silica particles beneath the surface. It was found that the accumulated tensile stress led to coating damage with the increase of laser pulse. The initial coating damage was observed to extend and interconnect to form large-area exfoliation. Splitting mechanism of SiO–Si TO_3_ was observed at vibration mode peaks of 1064 cm^−1^ and 1096 cm^−1^showing progressing irradiation damage. Based on this study, it would be helpful to suppress the damage probability of AR coating over KDP crystal applied in high-power laser systems. Moreover, the applicability of SNOM-PiFM method to study the Infrared Radiation (IR) spectra of ultra-thin coatings with transparent substrates was proposed.

## Introduction

High-power laser systems such as NIF, Nova, Omega in the USA, LMJ in France, Vulcan in the UK, and Gekko in Japan, were intensively studied recently to analyze indirect-drive of inertial confinement fusion, where extreme requirements on transmission and laser-induced damage threshold (LIDT) of laser coating was demanded^1–3^. In order to suppress the energy losses upon frequency-conversion component potassium dihydrogen phosphate (KDP) and potassium dideuterium phosphate (DKDP), antireflective (AR) coating with high transmission and LIDT at 355 nm was coated over their surfaces. Porous silica coating prepared by sol–gel method is a favorable candidate for high-power laser system due to its adjustable refractive index and considerable laser energy relaxation ability, which could endow the coating with excellent transmission (nearly 100% at 355 nm) and LIDT (~ 13 J/cm^2^ at 355 nm, 3 ns)^4–6^.

Blanching damages have been detected over this coating after irradiation with multi-pulse 355 nm laser beam at low flux (about 3 J/cm^2^) in vacuum, seriously deteriorating the transmission quality and lifetime of KDP component and resulting in component failure. Whitman^7^ reported coating damage over DKDP crystal under irradiation of 355 nm laser beam, in which blanching damage could be observed by microscope under vacuum but in air or nitrogen atmosphere. In addition, photo-emitting property and chemical structure of the coating surface was also studied in the paper, but further explanation of this phenomenon was not clearly stated. Transient absorption induced by 266 nm UV upon KDP/DKDP crystal has been discussed by Marshall^8^, and influence between this absorption and frequency-conversion efficiency has also been estimated. It was found that KDP/DKDP exhibited a broad transient absorption band (200–700 nm), which was attributed to dual-photon absorption of electrons. But whether this absorption was related to the blanching damage still remained unclear. Zhang^9^ studied the stress variation of porous silica coating over KDP crystal under heating and moisturizing process, showing that tensile or compressive stress might cause coating damage if critical conditions were exceeded. Pareek^10^ studied the effect of organic vapor on coating quality and LIDT, and the results showed that pollution could lead to the reduction of LIDT, and enhance the reflectivity of AR coatings, which provided useful information because the coating studied in this paper was applied in a vacuum chamber and organic contamination was inevitable.

Vibrational spectroscopy, such as Raman and infrared spectroscopy, are powerful tools for characterizing organic/semiconductor coatings. However, the coating thickness in our study is about 70 nm, which is far below the detection limit of traditional spectral characterization methods^11^. Scanning near-field optical microscope (SNOM) can solve this problem. It uses the probe of atomic force microscope to obtain the surface topography of the sample and apply infrared spectrum near the tip, which is similar to the effect of tip enhancement. In Photo-induced Force Microscope (PiFM) mode, the band 760 cm^−1^ to 1970 cm^−1^ has IR-like spectral characteristics; to a certain extent, it can be used as nano-FTIR to obtain a single spectral line on the sample surface or imaging the sample surface under a specific wave length. Rao et al.^12^ tested and compared the spatial and spectral resolution of IR-S-SNOM and AFM-IR of PDIF-CN_2_ polycrystalline films, and the results showed that, owing to the anisotropy of the crystal and the fundamental differences in how the methods work, the technique and polarization can result in significant spectral differences. Based on the near-field optical theory, Cui et al.^13^ studied the near-field characteristics of silver nanoparticles at the tip of metal atomic force microscope under the irradiation of fiber probe laser by using the finite element method. When the laser is transmitted in the fiber probe, the light of each mode gradually goes out, and the HE1 mode laser is left. The results show that the extreme point of tip enhancement occurs at the tip of AFM tip, the upper and lower part of Ag nanoparticles, and the upper and lower tip boundaries at the end of the conical fiber probe. In our study, the infrared properties of SNOM were used to compare the coating before and after laser damage occurred, to study the infrared properties of the coating under three states, and to analyze coating damage causes.

In the high-power laser system, large-area exfoliation of AR coating over KDP/DKDP was found to degrade the transmittance seriously and cause severe harm to the downstream optical components. Therefore, it is important to study the mechanism of coating exfoliation behavior. In summary, this type of laser coating damage is noticed recently and found very harmful for the transmission and lifetime of KDP/DKDP component, but studies aimed at this specific coating failure can be hardly found. In our previous work^14^, a series of structural changes in the coating damage process was analyzed, including the adhesion changes of the interface layer. It was found that the conversion behavior of Si–O–Si to Si–O–K at the interface during the damage process led to the enhancement of adhesion of the coating, which was very crucial to the coating damage. However, the properties of Si–O–Si bonds in the surface layer (such as bond length and bond Angle) during irradiation need to be studied and clarified. Thus, detailed study on structure, morphology, and stress variation of porous silica laser coating applied for high-power laser system was carried out in this paper, and the same coating without laser irradiation was also characterized as control reference. This work would facilitate the understanding of coating damage mechanism and offer useful information to suppress the failure probability of frequency-conversion component for high-power laser system.

## Methods

### Materials

Tetraethylorthosilicate (TEOS) was purchased from Sinopharm Chemical Reagent Co. Ltd. (Beijing, China). Hexamethyldisiloxane (HMDS) was purchased from Shanghai Aladdin Bio-Chem Technology Co., Ltd. (Shanghai, China). Anhydrous ethanol and ammonia water were purchased from Chengdu Kelong Chemical Co., Ltd. (Chengdu, China). The water was deionized. The TEOS were distillation purified before use. Other chemicals were used without further purification.

### Preparation of colloidal silica sol

**Figure 1:**
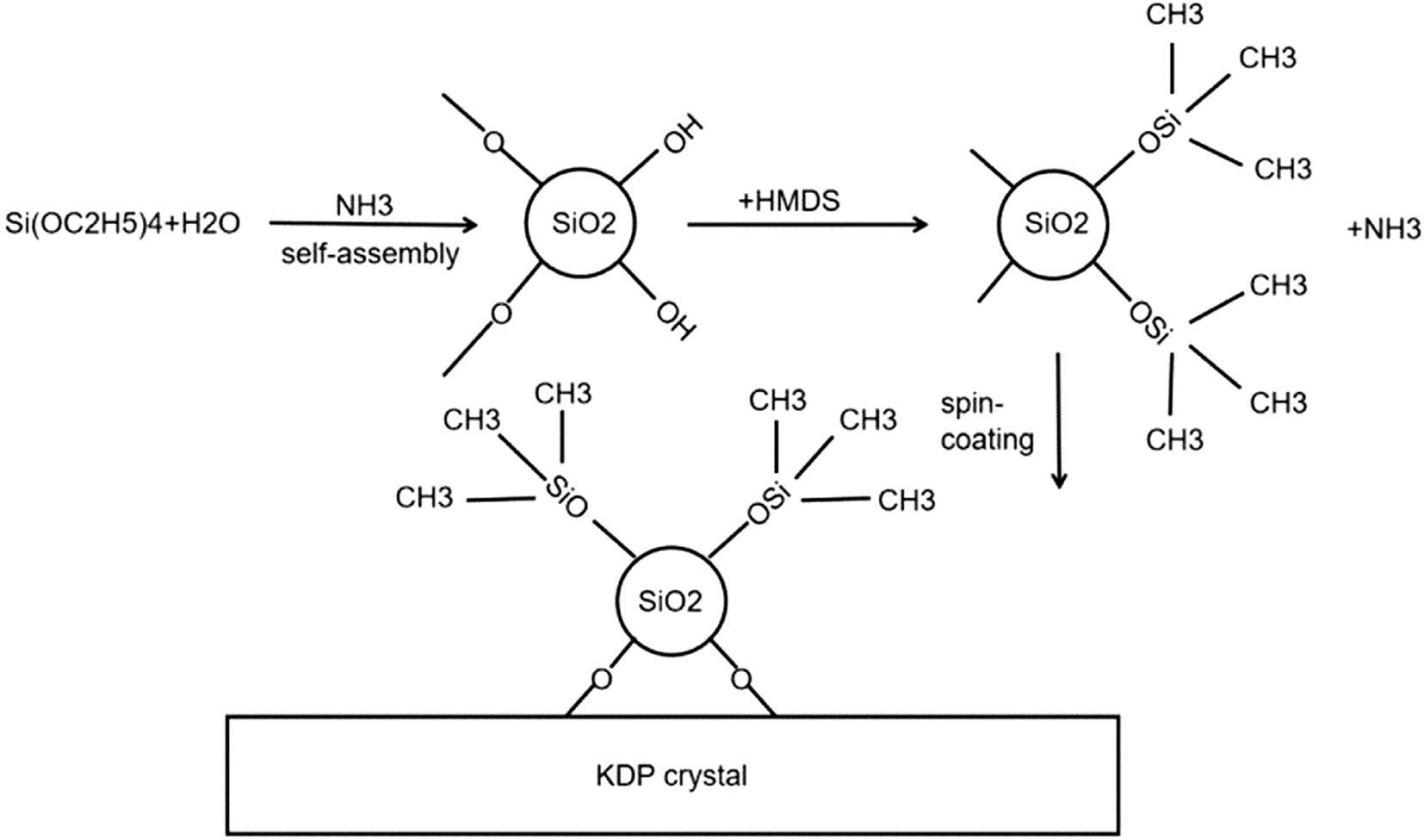
Schematic diagram of the preparation process of silica sol drawn using Microsoft™ Visio Professional 2021 (https://www.microsoft.com/en-us/microsoft-365/p/visio-professional-2021/cfq7ttc0hgxw?activetab=pivot:overviewtab).

The silica colloidal suspension was prepared by Stöber method^15^, in which TEOS (208 g) was used as matrix and mixed with anhydrous ethanol (1610 g) and ammonium hydroxide aqueous solution (51.4 g, containing 30% ammonia) with stirring at 6 °C for 3 h. The solution was kept still in a sealed glass container for three to five days at room temperature to implement the aging process. It was then refluxed for 10 h to remove the extra ammonia. This colloidal suspension contained about 3.3% silica by weight in ethanol and was filtered through a 0.2-µm polyvinylidene fluoride (PVDF) membrane filter prior to use. The HMDS (180 g) was added into the resulting colloidal silica sol (1818 g, 3.3 wt%), then stirring and aging were performed at room temperature for another 7 days, in order to partially replace –Si(OH) with –Si(CH_3_)_3_ to increase the hydrophobic property (Fig. 1).

### Preparation of sol–gel coatings

For KDP/DKDP variable frequency crystals, a matching coating system is required to meet the conversion requirements from 1 ω (1064 nm) to 3 ω (355 nm)^16^, and it is found that damage often occurs over the coating for 3 ω. Therefore, the sol prepared in “Preparation of colloidal silica sol” section was spin-coated over KDP crystals (50 mm × 50 mm × 10 mm) under a velocity of about 300 rpm at a rated time of 80 s to modulate the coating thickness to the designed value (~ 70 nm)^17–20^. The test results of coating thickness and laser damage threshold can be found in literature^14^. The resulting coating was used as reference (named as coating A). On the other hand, irradiated coating (named as coating B) was prepared using the same method as coating A and applied in the high-power laser system, which suffered 200–300 pulses of 355-nm laser (2–3 J/cm^2^, 3 ns).

### Characterization of coatings

Dynamic profiler (4D NanoCam Sq) was employed to evaluate the height gradient of coating. Using a 10 × lens to focus on the sample surface, the roughness and depth profile information of the coating surface can be obtained within a range of about 1 cm^2^, which can be used to evaluate the depression depth of the damaged part of the coating surface. Surface morphology of coating was characterized using SEM (ZEISS Gemini 300). Profilometer (Dektak 150) was utilized to estimate the curvature height of coating, based on the path of probe scratching over the sample surface within a distance of 2 mm. The corresponding curvature radius (R_c_) can be derived according to the curvature height, and stress (σ) information can be calculated based on the R_c_ results in association with Young’s modulus and Poisson ratio of KDP crystal.

**Figure 2:**
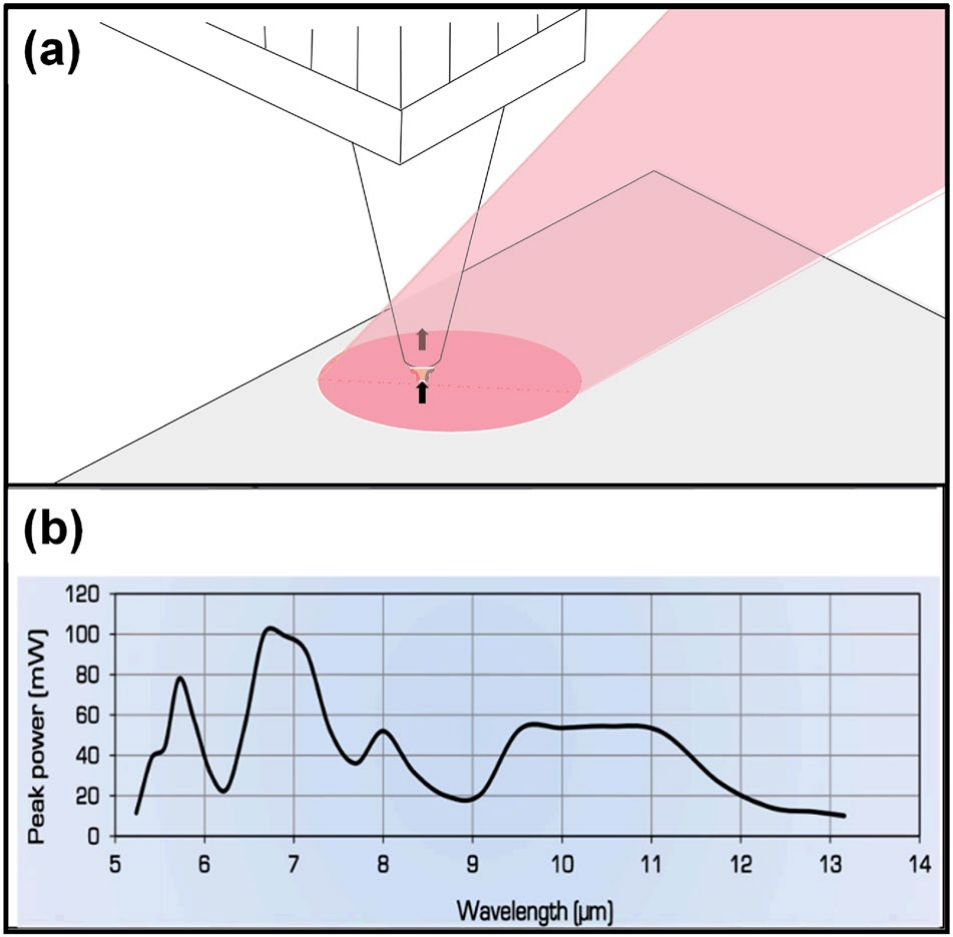
Information of SNOM-PiFM method, (**a**) schematic diagram drawn using Visio™ Professional 2021, and (**b**) power spectrum of laser.

The Anfatec Instruments/VistaScope SNOM mode was based on the AFM characterization, with addition of an IR module. Laser is irradiated at the tip of the needle to enhance the characterization capability. Figure 2a shows the principle diagram of SNOM-PiFM. An AFM tip is used to mechanically detect the force interaction between the tip and sample due to laser excitation. Photo-induced force microscopy (PiFM) and spectroscopy (PiF-IR) can perform complete chemical analysis down to resolutions of less than 10 nm. PiF-IR spectra can be used to identify materials and find unique IR bands for imaging. Fixed wavenumber PiFM images can be used to create chemical maps of the surface. The IR spectrum as well as the surface topography information can be obtained from the tip, and the scanning range is 1 μm^2^. The infrared spectral information of the coating in the 760–1970 cm^−1^ band was recorded using this method with certain laser power given in Fig. 2b.

## Results and discussion

### Macroscopic feature

**Figure 3:**
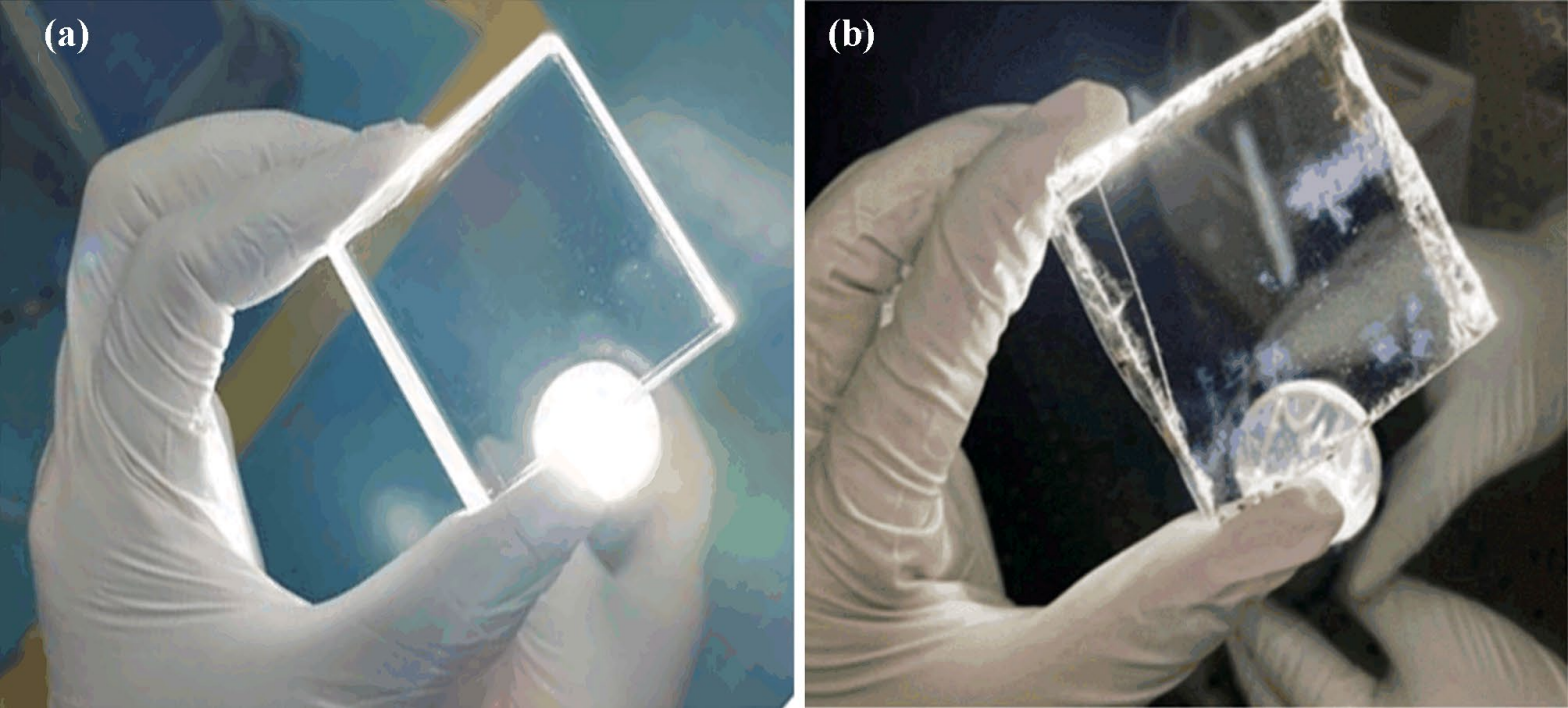
Images of coating under flashlight, (**a**) coating A, and (**b**) coating B.

**Figure 4:**
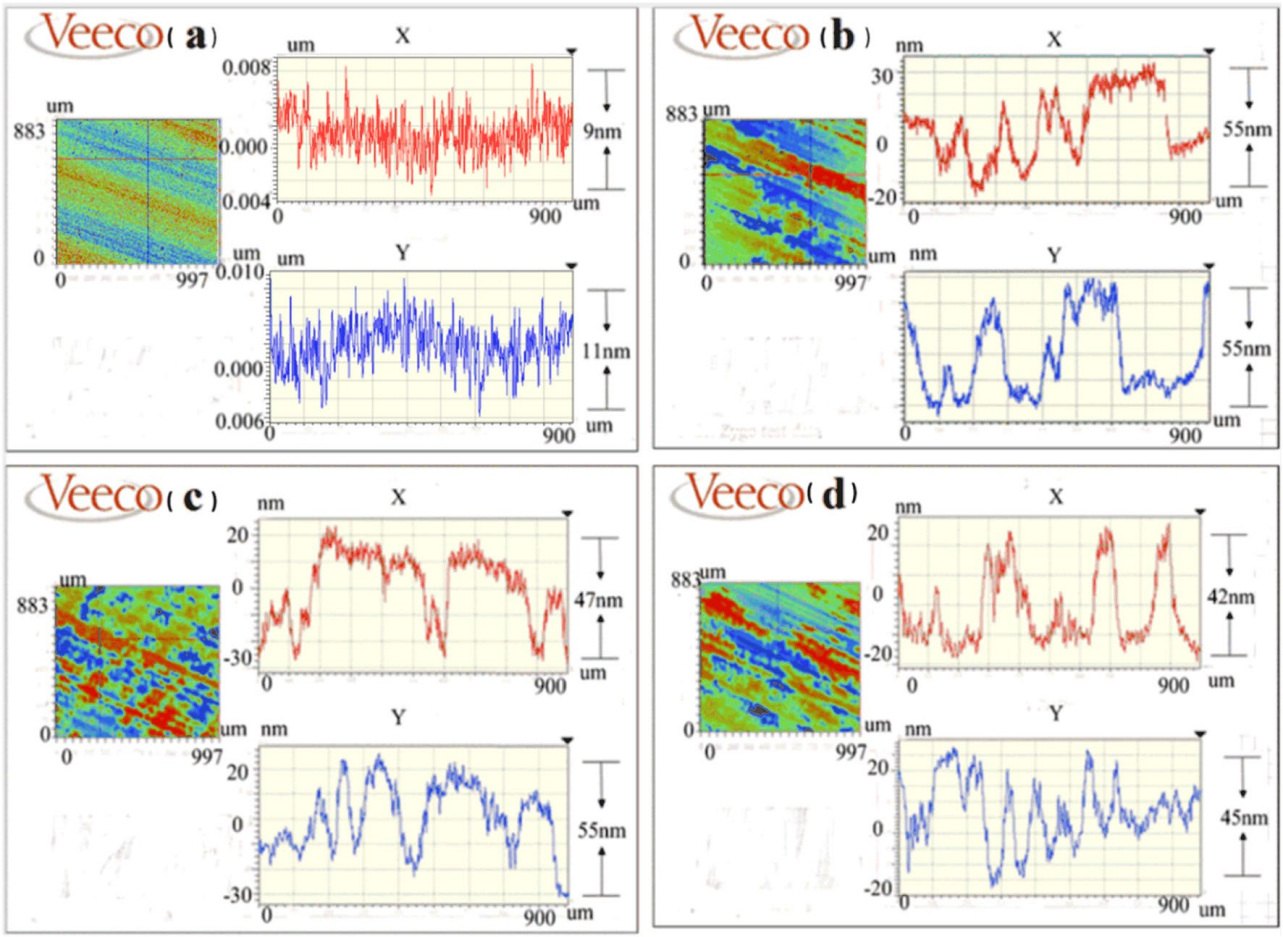
Surface topography of coatings characterized using optical profiler, (**a**) coating A, (**b**)–(**d**) different damage places at coating B.

Figure 3 was taken by us in our cleanroom and shows the typical shapes of two different coatings under flashlight, in which the surface of coating A is smooth, and no visible defects can be observed. However, irregular whitish damages are found randomly distributed over the surface of coating B. These two coatings were characterized by an optical profiler, and the results are shown in Fig. 4. The surface fluctuation of coating A is not significant, while the surface of coating B presents a step configuration with a differential between the highest and the lowest step of about 50 nm. According to the analysis results of optical profiler, the whitish parts are predicted to be coating exfoliations, with height difference ranges from 20 to 60 nm. Because the coating thickness is designed to be 70 nm, most of this whitish exfoliation is not enough to uncover the substrate surface; thus, most of the coating still adheres to the crystal surface after coating degradation and exfoliation.

### Microstructure

**Figure 5:**
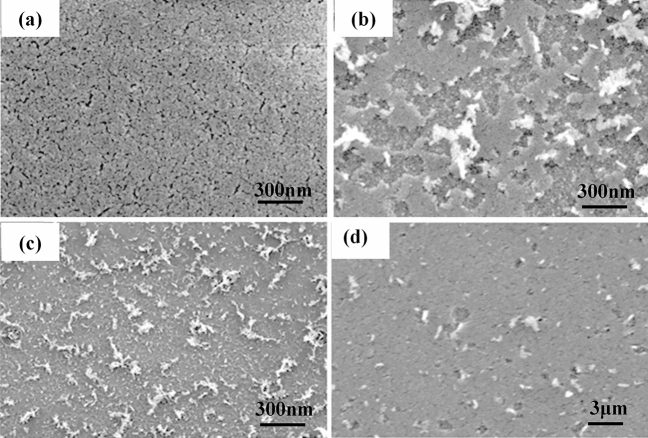
SEM morphology of coatings, (**a**) coating A, (**b**) coating B, (**c**) whitish part in coating B at lower magnification, (**d**) unbroken part in coating B with 50,000 times magnification.

Microscopic morphology of coatings characterized by SEM is shown in Fig. 5. In Fig. 5a it can be seen that the silica particles in coating A are densely arranged with some microcracks over the surface, whereas whitish exfoliations are clearly observed in coating B, displayed in Fig. 5b. In addition, nanoparticles still can be found beneath the exfoliated coating, which indicates that the bottom layer is not KDP substrate but silica coating closer to the KDP substrate. This conclusion is consistent with the observations made with the optical profiler. SEM images of whitish parts over coating B at lower magnification are shown in Fig. 5c, where random arrangement of the whitish parts is further observed. In addition, SEM image of unbroken area of coating B with a 50,000 × magnification is shown in Fig. 5d. Only a few pits and white particles are observed in the unbroken area, and the size range of these pits is from tens to hundreds of nanometers. This is possibly the initial state of overall whitish damage. When this microscopic damage further intensifies into a scale, it can be manifested as a visible whitish damage.

The SEM results provide a reasonable explanation for the macroscopic phenomenon of whitish exfoliation over coating B. In the process of 355 nm laser irradiation, many nano-scale pits will be generated over the coating due to stress expansion caused by heating or internal defects^21^. The size of this kind of pits grows bigger with the increase of laser pulse, leading to the formation of large-area coating exfoliation. The exfoliated part adheres to the coating surface and causes visible whitish spots. It can be speculated that the cause of this damage may be related to the combined effect of organic pollution and laser irradiation. Occupation of coating micropores by organic pollutants reduces the quality and performance of laser coating, resulting in gradual loss of the ability to release high-power laser energy.

### Calculation of residual stress

**Figure 6:**
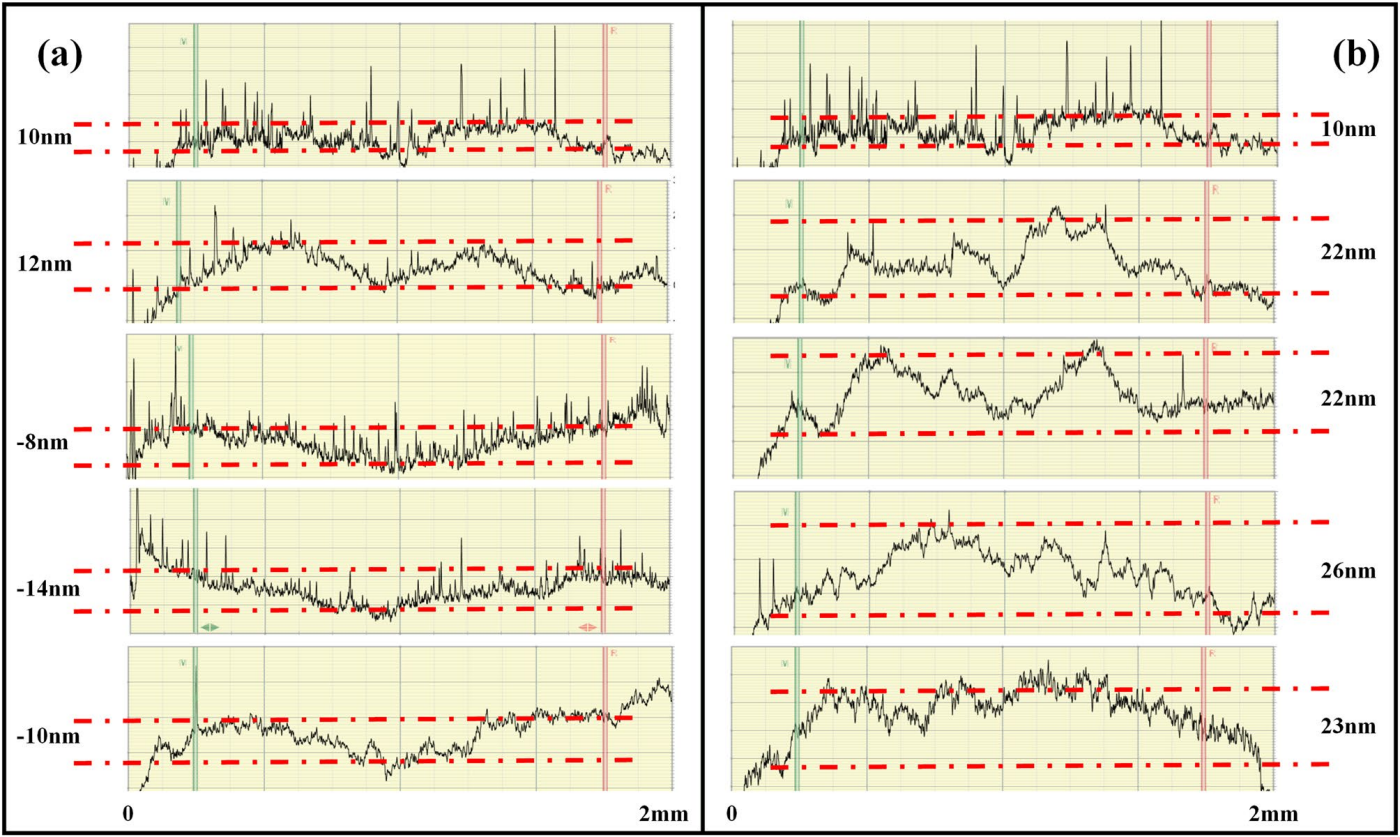
Profilometer measurement results of curvature variation, (**a**) coating A, and (**b**) coating B.

The coating can be regarded as the upper surface of KDP substrate since the coating thickness (~ 70 nm) is significantly smaller than that of substrate (~ 10 mm). Therefore, the stress of coating can be calculated based on its bending^22^. The curvature was measured using a Dektak 150 probe profilometer, with scan length about 2 mm. Figure 6 shows five sets of typical curvature height of each coating, residual stress (*σ*_*r*_) and radius. The residual stress (*σ*_*r*_) and the radius (*R*) can be calculated by Eqs. (1) and (2), respectively^23^.

1 $$ {\upsigma }_{r} = \frac{1}{6}\left[ {\frac{1}{{R_{post} }} - \frac{1}{{R_{pre} }}} \right]\frac{{E_{s} }}{{\left( {1 - v} \right)}}\frac{{t_{s}^{2} }}{{t_{f} }} $$

2 $$ {\text{R}} = \frac{{L^{2} }}{8B} $$

where *R*_*pre*_ and *R*_*post*_ are the radius of curvature before and after treatment (*R*_*pre*_ was approximated as + ∞), *E*_*S*_ and *ν* are the modulus (KDP crystal [001] is 40 GPa) and Poisson's ratio (KDP crystal [001] is 0.24) of KDP substrate^24,25^, *t*_*s*_ and *t*_*f*_ are the thicknesses of substrate and coating, respectively, and *L* and *B* are the scanning length and curvature height of the tested sample, respectively. Assuming that the scan length *L* of the sample is much greater than the curvature height *B*, the radius *R* can be calculated using Eq. (2).

Generally, *σ*_*r*_ can be attributed to three different factors: (1) inherent stress during coating formation, (2) stress caused by the mismatch of thermal expansion coefficient between substrate and coating, (3) stress caused by the absorption of foreign molecules^24,25^. The results of *σ*_*r*_ estimated based on *R* are listed in Table 1, where positive and negative signs represent bumps and depressions along the surface of the base, respectively. It can be seen that the inherent stress in the coating formation process is mainly compressive stress. After multi-pulse interaction of ultraviolet laser irradiation, the *σ*_*r*_ of coating B turns into tensile stress, which might be caused by thermal expansion and result in whitish damage of the coating.

**Table 1:** Residual stress estimation results of coating A and coating B.

	Coating A	Coating B
Average height (nm)	− 2	11.26
Radius of curvature (m)	− 156250	27753
Residual stress (GPa)	− 0.08	0.45

### Coating analyzed by SNOM-PiFM infrared spectrum

#### Coating infrared spectrum

Because traditional infrared spectroscopy detection is affected by coating thickness and matrix reflection, the infrared spectral information of coating in our study cannot be obtained even if total reflective infrared spectroscopy is used. However, this problem can be solved by detecting infrared spectra based on the enhanced tip of atomic force probe, which combines the traditional infrared spectroscopy and atomic force probe. Here, the spectral information of the specified tip region was measured on the IR spectrum in three typical areas; coating A, whitish part of coating B, and unbroken part of coating B.

**Figure 7:**
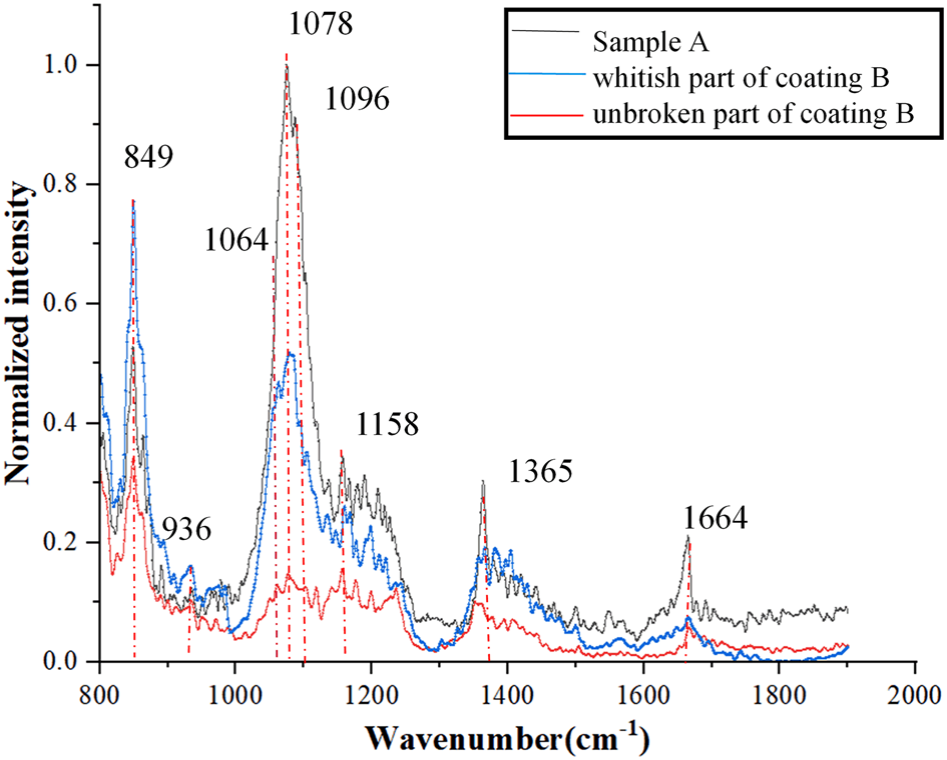
Infrared spectral results of three samples.

In the experimental process, it was found that the coating was easy to adhere to the surface of the probe, it was difficult to obtain a relatively complete coating morphology, and the scanning area could not be carried out. Therefore, we used the method of averaging multiple measurements of a single sample to obtain specific infrared spectral information. The infrared spectrum measurement results of the three samples are shown in Fig. 7. The abscissa is wave number and the ordinate is the normalized intensity; the graphic reflects the intensity of the absorption peak.

Table 2 lists the peak position of coating A and damaged part in Coating B between 800 and 1900 cm^−1^, which is the main range for the analysis of the Si-based structure^26,27^. As for the unbroken part in Coating B, it is found that all the fingerprint peaks are not obvious. Due to the small surface detection limits (~ 10 nm) of SNOM-PiFM, it is assumed that the microstructure over the surface of unbroken part in Coating B is already destructed after laser irradiation, although the surface morphology seems to be complete. Therefore, the IR spectrum could not recognize any of the Si-based signal. However, the damage part in Coating B exposes some fresh coating structure while its surface is exfoliated, which can still be recognized via SNOM-PiFM. Therefore, the difference between Coating A and damaged part in Coating B is discussed here. The absorption peak near 849 cm^−1^ represents the vibration of Si-CH_3_, and the absorption peak near 936 cm^−1^ represents the stretching vibration of Si–OH. The absorption peak of 1078 cm^−1^ represents the asymmetric ω_4_ (TO_3_) extended vibration of Si–O–Si bond. The absorption peak near 1158 cm^−1^ represents the longitudinal optical mode ω_2_ (LO_3_ mode) of SiO_2_ high frequency vibration. The absorption peak near 1365 cm^−1^ represents the shear bending vibration of H–O–H. The absorption peak at 1664 cm^−1^ may be caused by residual organic solvents such as ethanol^28^.

**Table 2:** Coating peak position, intensity, and band assignations of two states.

Position (cm^−1^)	Intensity of coating A	Intensity of damaged part in coating B	Band assignations
849	Strong	Strong	Si–CH_3_ bending
936	Strong	Strong	Si–OH Stretching vibration
1064	weak	weak	TO mode for tetrahedral SiO_2_
1078	Strong	weak	TO_3_ mode for tetrahedral SiO_2_
1096	weak	weak	TO mode for octahedral SiO_2_
1158	Strong	weak	LO_3_ mode for octahedral SiO_2_
1365	Strong	weak	H–O–H shear bending vibration
1664	Strong	weak	Residual organic solvents

#### Vibration mode splitting analysis of Si–O–Si

Among these absorption peaks, ω_4_ (TO_3_) is the main characteristic peak of SiO_2_, and the variation of this peak reveals the change of structure and characteristics of SiO_2_. Here, the main characteristic absorption peaks of SiO_2_ in the damaged coating show two frequency shifts in opposite directions, one to the low frequency region (1064 cm^−1^) and the other to the high frequency region (1096 cm^−1^). This phenomenon is considered to be related to the change of porosity of sol–gel coating.

**Figure 8:**
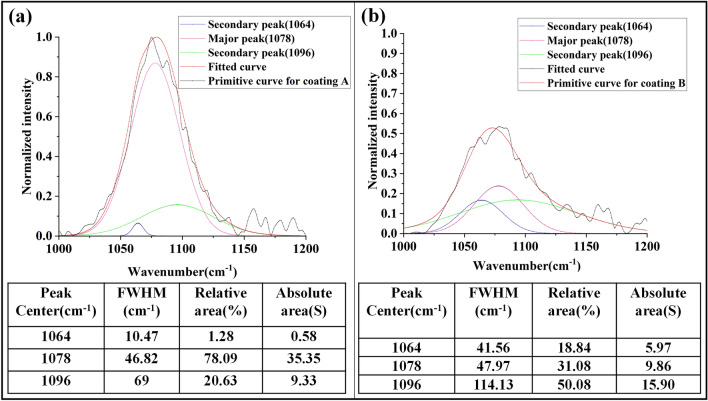
Peak fitting results of different samples, (**a**) coating A, and (**b**) damaged part of coating B.

Figure 8 shows the peak fitting results of coating A and the damaged part of coating B in the range from 1000 to 1200 cm^−1^. It can be seen that the relative area of the main peak of coating A in the initial state at 1078 cm^−1^ is 78.01%. The relative area ratios of the two secondary peaks (1064 cm^−1^ and 1096 cm^−1^) are 1.28% and 20.63%, respectively, which indicating that there is almost no densification of the coating in the initial state, but there is some initial residual stress. In Fig. 8b, the relative area of the main peak of coating A at 1078 cm^−1^ decreased to 31.08%, while the relative area ratio of the two secondary peaks (1064 cm^−1^ and 1096 cm^−1^) increased to 18.84% and 50.08%, respectively, indicating that there exists a shrinking Si–O–Si bond inside the damaged area. There are also Si–O–Si bonds which are stretched due to stress accumulation. On the other hand, the sum of the absolute contents of the three peak areas in Fig. 8b (31.73) is also lower than that in Fig. 8a (45.26), which implies that irradiation damage is accompanied by the fracture of part of Si–O–Si bond.

**Figure 9:**
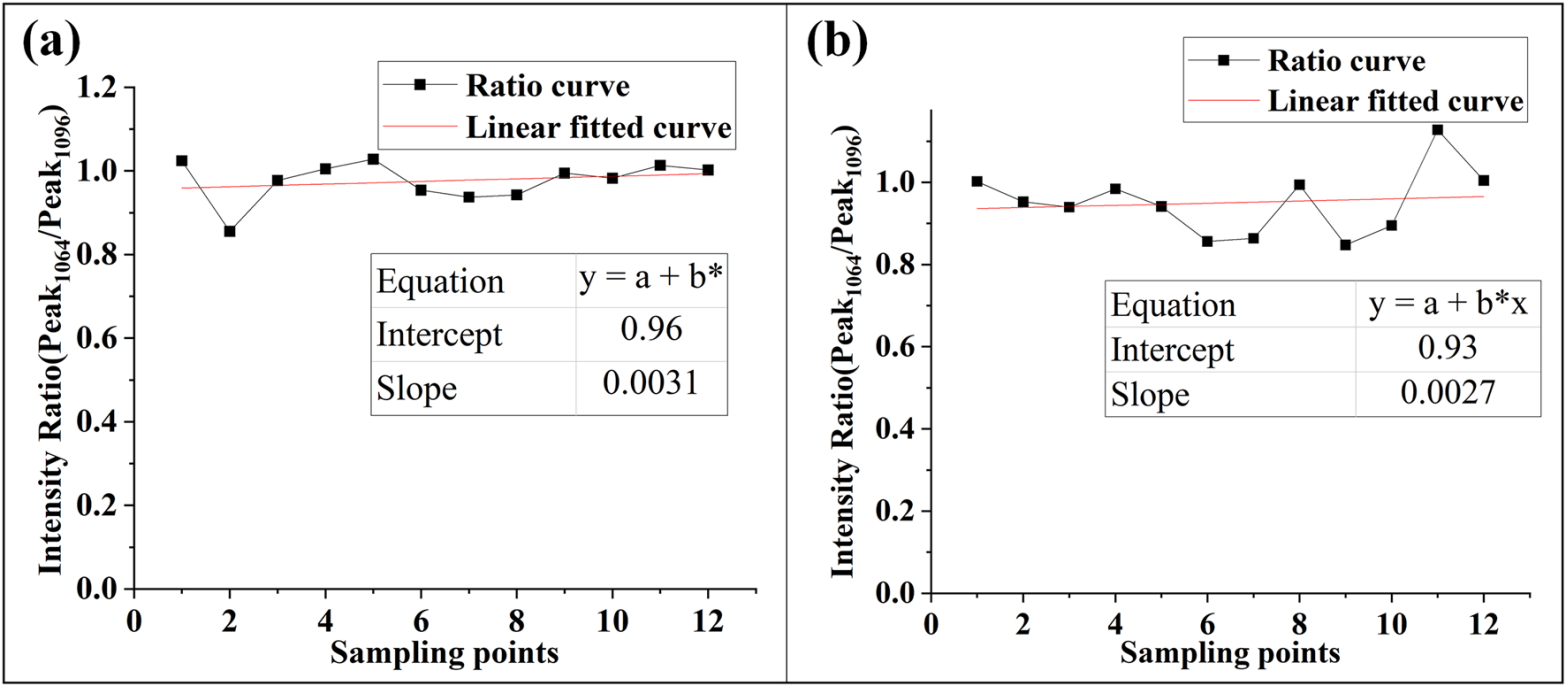
Intensity ratio distribution of Peak_1064_/Peak_1096_, (**a**) in Coating A, and (**b**) in damaged part of coating B.

In order to study the formation of the splitting peak, two splitting peaks around 1078 cm^−1^ were compared. Figure 9 shows the intensity ratio of Peak_1064_/Peak_1096_ in the Coating A and damaged part of Coating B, and the ratio curve is fitted with 12 sampling points. It can be seen that the graph of vibration ratio Peak_1064_/Peak_1096_ is almost a horizontal line (slope 0.0031 and 0.0023), and their ratio is close to 1:1. Nakamura et al.^29^ reported in detail the relationship between the frequency shift of ω_4_ (TO_3_) peak of Si–O–Si and the stress change. They suggest that the low-frequency displacement of the ω_4_ (TO_3_) peaks is related to dehydration during thermal processes, while the high-frequency displacement is related to the rearrangement of Si and O atoms. Combined with the above SEM study, we believe that the complete coating corresponds to Peak_1064_ in infrared spectrum, while the location of spalling damage pit corresponds to Peak_1096_. As shown in Table 2, when the peak position changes from 1078 to 1096 cm^−1^, the coordination number of silicon atom changes correspondingly, from Si–O tetrahedral structure to octahedral structure. The macroscopic bleaching phenomenon is caused by the increase of scattering caused by bond length stretching at peak_1096_. In addition, the study in “Calculation of residual stress” section provides the stress measurements of the coating after irradiation. The coating exhibits tensile stress at macro level, while the shrinkage effect of the coating shows the movement of infrared spectrum towards short-wavenumber direction.

The change of ω_4_ (TO_3_) absorption peak reveals the evolution of SiO_2_ coating structure. According to the theory of Sen, Thorpe^30^ and Galeener^31^, TO_3_ asymmetric tensile frequency ω_4_ is a function of Si–O–Si bridge angle *θ* and Si–O tensile force constant. The change of peak frequency means the change of average Si–O–Si bridge angle. Because the average Si–O–Si bridge angle *θ* has the following relationship with the peak frequency:

3 $$ {\upomega }^{2} = \frac{\alpha }{{m_{O} }}\left( {1 - cos\theta } \right) + \frac{4\alpha }{{3m_{si} }} $$

where *ω* is the frequency of peak ω_4_ (TO_3_), *α* is the tensile force constant of Si–O–Si bond, and *θ* is the average bridge angle of Si–O-Si bond; *m*_*O*_ and *m*_*Si*_ are the masses of oxygen and silicon atoms, respectively. According to the above formula, the peak frequency of ω_4_ (TO_3_) increases with the increasing of Si–O-Si bond angle. Conversely, as the bond angle of Si–O-Si decreases, the peak frequency of ω_4_ (TO_3_) also decreases. According to our previous studies, coating damage is not a single process, but a series of complex processes such as coating densification, coating thermal stress accumulation and coating damage. Even if the coating is damaged, the undamaged part is included. Therefore, the shift of ω_4_ (TO_3_) peak to the lower wave-number direction is related to the structure with smaller siloxane ring, larger Si–O-Si bond angle and more porous Si–O-Si bond length. The increase of Si–O-Si bond angle and Si–O bond length leads to the decrease of tensile force, which corresponds to the accumulation of thermal stress during laser failure. On the contrary, the shift of ω_4_ (TO_3_) peak to higher wave-number direction represents the contraction of Si–O-Si bond angle and the shortening of Si–O bond length, corresponding to the densification process of coating during laser damage.

Besides the vibration of Si-CH_3_ at 849 cm^−1^ and the stretching vibration peak of Si–OH at 937 cm^−1^, all wave numbers decreased to different degrees, which may be related to the evaporation of organic solvents and water molecules during the irradiation process.

Based on the above discussion, the laser irradiation damage process of coating is summarized as follows:

1. Most of the main structures in the coating will be damaged by initial irradiation, and their intensity will decrease at the same time. This damage originates from the surface, which can be obtained from the undamaged part of coating B in Fig. 7.

2. The cumulative subsequent irradiation will bring two results: Firstly, it will cause the contraction of Si–O–Si bond angle, and the fingerprint peak of Si–O–Si bond in the coating (1078 cm^−1^) will shift to 1064 cm^−1^, which is consistent with the densification effect of laser irradiation in vacuum. On the other hand, the coating undergoes ultraviolet irradiation in vacuum, and the residual stress brought by each pulse will be retained in the coating. Therefore, a part of Si–O–Si bond will undergo bond angle stretching with the accumulation of such residual stress, and the fingerprint peak of Si–O–Si bond (1078 cm^−1^) on the chemical bond will shift to 1096 cm^−1^. This point corresponds to the residual stress calculation conclusion obtained in “Calculation of residual stress”section.

3. The final cause of coating damage may be the accumulation of residual stress from irradiation, because the coating densification effect only changes the porosity and transmittance of the coating, not enough to cause catastrophic damage.

### Damage mechanism

Damage of coating properties observed in SEM images follows two trends. One is that the coating is still in the undamaged state adsorbed on the surface, which is obviously denser than the intact state and lacks the porous structure of original morphology. The other is the surface damage or surface suspension state of strip steel, which is a whitish damage at macro level. These two states correspond to the peak and valley bottom part of the coating profile (40–55 nm). Meanwhile, these two states of coating correspond to two frequency shift states of Si–O–Si bond in PiFM. The first image shows the coating shrinking and the ω_4_ (TO_3_) peak moving to the low-frequency direction. The second image shows the coating stretching, with the ω_4_ (TO_3_) peak moving towards high-frequency. The stress change experiment of the coating corresponds to the Si–O–Si bond moving to the high-frequency direction in PiFM.

The LIDT of traditional sol–gel porous silica coating applied to KDP crystal is commonly high (~ 13 J/cm^2^ at 355 nm, 3 ns), and it is rare to observe such white spall damage with naked eyes at the irradiation energy below the damage threshold. However, under the condition of low-flux multi-pulse 355-nm laser and vacuum irradiation, the coating showed a huge spalling of hundreds of nanometers, resulting in a large area of whitish damage. According to the current research conclusions, the damage mechanism of the coating can be explained as follows: under laser irradiation, Si–O–Si bond length in the coating shrinks, ω_4_ (TO_3_) peak shifts from 1078 to 1064 cm^−1^, which makes the coating structure denser and porosity lower. This process does not cause catastrophic damage to the coating, but it sets the stage for subsequent damage. Laser irradiation produces thermal expansion effect at macro level and increases with the accumulation of thermal stress. Meanwhile, residual stress accumulates inside the coating, which reduces the radius of curvature of the coating from 83 to 18.23 m. Some Si–O–Si bonds increase with this process and ω_4_ (TO_3_) peak shifts from 1078 to 1096 cm^−1^, which is accompanied by the change of coordination number of Si atom from tetrahedral structure to octahedral structure, resulting in increased scattering and macroscopic whitening. Eventually, catastrophic coating damage occurs.

## Conclusions

The damage behavior of sol–gel silica coating from KDP crystal applied for high-power laser system was characterized by means of instruments, and its residual stress variation was calculated. Reasonable explanation of this phenomenon is proposed based on the detailed characterization results. It is considered that the damage behavior is accompanied by the ω_4_ (TO_3_) vibration mode of Si–O–Si bond splitting in two opposite directions. This behavior degrades the relaxation ability against laser energy of coating, and stress accumulation is generated under multi-pulse laser interaction. Nano-scale initial microscopic damage is first induced and gradually expanded and interconnected with each other, to form macroscopically visible damage with the increase of laser pulse, which eventually results in failure of the entire KDP component.

## Data Availability

The data that support the findings of this study are available from the corresponding author upon reasonable request.
